# Assessment of Right Ventricular Function, Blood Lactate Levels, and Serum Peptidomics Profiles Associated With Mitral Valve Disease in Dogs

**DOI:** 10.3389/fvets.2021.789137

**Published:** 2022-01-20

**Authors:** Soontaree Petchdee, Mona Yalong, Methawee Kaewnet, Burasarin Ithisariyanont, Tanawat Padawong

**Affiliations:** Department of Large Animal and Wildlife Clinical Sciences, Faculty of Veterinary Medicine, Kasetsart University, Bangkok, Thailand

**Keywords:** canine, echocardiography, heart failure, lactate, peptide profile

## Abstract

**Background:**

Degenerative mitral valve disease is a common heart problem in dogs. The aims are to evaluate the relationships between right and left ventricular function, and blood lactate concentrations, assess prognostic contribution, and investigate whether serum peptidomics profile could reveal markers or determine the stage in dogs with valve degeneration.

**Materials and Methods:**

Ninety-three dogs were evaluated in this study. Thirty-nine dogs' serum was collected and analyzed using matrix-assisted laser desorption/ionization-time of flight mass spectrometry. The Kaplan–Meier curve was used to predict the outcomes of mitral valve disease. Follow-up was obtained by a questionnaire or telephone to determine a survival time.

**Results:**

The BUN/creatinine ratio, vertebral heart score, and left atrium/aorta ratio were the independent predictors of cardiac mortality. Right ventricular systolic dysfunction was found in 50% of dogs with mitral valve disease. Dogs with right ventricular dysfunction had a significantly higher incidence of lower fractional shortening and larger right ventricular dimensions. The occurrence of right-sided dysfunction is proportionate to age and the degree of left ventricular dysfunction. High blood lactate concentrations were investigated in dogs with mitral valve disease stage C compared with stage B. The peptides such as mitogen-activated protein kinase, kallikrein, and tenascin-C appeared in the heart disease progression group.

**Conclusion:**

Right-hearted function assessment, blood lactate levels, and peptidomics analysis may help early detection and prognosis of this disease in dogs. Peptidomics profiles from this study demonstrate the possibility for prognosis indicators of heart valve degeneration.

## Introduction

Left-sided congestive heart failure is commonly associated with mitral valve degeneration and is a significant heart problem in dogs (1, 2). Left ventricular function plays a vital role in evaluating dogs with congestive heart failure, while the right ventricle function has been less studied (3). The American College of Veterinary Internal Medicine (ACVIM) consensus statement has been used to classify dogs to determine the severity of this disease. Dogs have been classified into stages A, B, C, and D (4, 5). High blood lactate concentrations are frequently observed in dogs with abnormal thoracic radiographs such as cardiomegaly and pulmonary parenchyma (6). Dogs with degenerative mitral valve disease often have impaired renal function. Renal dysfunction is the critical determinant of cardiac compensation and disease progression (7).

Right ventricular function is an essential determinant of a human's clinical status with chronic heart failure (8). The right ventricle is commonly affected by cardiac diseases such as tricuspid dysplasia and dilated cardiomyopathy. Previous studies suggested a close relationship between right and left ventricle dysfunction. However, the right-sided functional assessment remains challenging due to the right heart anatomy (9–11). Echocardiographic measurement is a non-invasive method for heart function assessment. Echocardiographic imaging of dogs with left-sided congestive heart failure has been associated with adverse cardiovascular outcomes such as reduced cardiac output and decreased contraction function (12, 13). Right-hearted dysfunction has been verified in many human cardiac diseases, and clinical evaluation of right-sided heart function has also been reported in animals (14–16). Many parameters, such as right ventricle fractional area change (FAC), myocardial velocity of the lateral tricuspid annulus (S′), and tricuspid annular plane systolic excursion (TAPSE), are correlated well-with the right heart function.

Proteome analysis is a novel tool for exploring the changes in protein composition, and proteomics network analysis has been widely used to identify protein biomarkers in many diseases (17). It has shown that circulating proteins associated with heart failure can identify the stage increased across the heart failure process (18). A previous study identified phosphorylation of p38 mitogen-activated protein kinase (p 38 MAPK) and suggested linking p38 to inflammation, cell cycle, apoptotic process, and cell differentiation. In addition, the signaling mediated by the MAPK pathway may play a role in cardiac injury and remodeling (19). Many studies reported that the use of protein infusions such as kallikrein could improve cardiac function and reduce cardiomyocyte apoptosis and reduce inflammatory cell accumulation in the heart after myocardial ischemia (20, 21). Tenascin-C (TN-C) was also another peptide found in the serum of heart failure patients and suggested using it to predict ventricular remodeling and poor prognosis (22). The proteomic network assessment might be allowed in the evaluation of therapeutic targets for valvular heart diseases.

The present study aimed to assess the prognostic information about valvular heart disease in dogs, examine the echocardiographic alterations, assess the right ventricular function associated with mitral valve disease, determine the blood lactate concentrations, and MS-based identification of proteins and protein interacting in the mitral valve degeneration dogs.

## Materials and Methods

### Animals

This study was performed in clinical cases at Kasetsart University Veterinary Teaching Hospital Kamphaeng Saen from January 2019 to April 2020 (Table 1). The follow-up was obtained by a questionnaire or telephone to determine a survival time. The Ethics Committee approved all procedures in the study for Animal Experiments, Kasetsart University. The owners provided informed consent before enrollment. Ninety-three dogs with mitral valve degeneration were recruited into the study. The breeds included Poodle, Shih Tzu, Pomeranian, and Crossbreed; body weight ranged from 2 to 20 kg. All dogs had normal or unremarkable hematology and chemistry before enrollment and had not previously received oral positive inotropic agents. Each dog showed cardiac murmur and abnormal cardiac dimension from chest radiography. The owners provided informed consent before enrollment. Dogs demonstrated evidence of the clinical signs of stages B and C according to the ACVIM classification system. The MMVD B1 group included 24 dogs, and stage B2 (37 dogs), dogs affected by MMVD stage B1 and B2 with no clinical signs of congestive heart failure (asymptomatic). No radiographic evidence of congestive heart failure and no evidence of pulmonary edema were detected, but there was sufficient hemodynamic change to cause echocardiographic evidence of left atrial dilatation with an LA/AO ratio >1.6 in stage B2. The MMVD C group included 32 dogs affected by MMVD with echocardiographic evidence of mitral valve leaflet thickening and regurgitation. Dogs in stage C showed clinical signs of congestive heart failure identified by clinical examination and radiographic evidence of pulmonary edema. The survival information was examined from telephone calls and analyzed as the time between the first visit for echocardiographic recording and the date of death.

**Table 1:** Characteristics and echocardiographic variables in 93 dogs.

**Parameters**	**Mean ±SEM**
**ACVIM classification [number (%)]**	
Stage B	65.6
Stage C	34.4
VHS	11.54 ± 1.43
LA/AO ratio	1.59 ± 0.05
EDV (ml)	40.75 ± 3.19
ESV (ml)	17.35 ± 1.83
LVIDd (cm)	3.04 ± 0.10
LVIDs (cm)	2.05 ± 0.09
E deceleration time (ms)	107.37 ± 3.29
FS (%)	28.07 ± 0.94
**RV parameters**	
RV basal diameter (cm)	1.22 ± 0.05
RV mid diameter (cm)	0.93 ± 0.04
RV longitudinal diameter (cm)	2.27 ± 0.09
FAC (%)	29.86 ± 1.44
TAPSE (cm)	1.22 ± 0.06
**TR [number (%)]**	
Mild	60
Moderate	40

*VHS, vertebral heart score; LA/AO ratio, left atrium/aorta ratio; EDV, end-diastolic volume; ESV, end-systolic volume; LVIDd, left ventricular internal diameter during diastole; LVIDs, left ventricular internal diameter during systole; FS, fractional shortening; RV, right ventricle; FAC, fractional area change; TAPSE, tricuspid annular plane systolic excursion; TR, tricuspid regurgitation. Data are shown as mean ± SEM*.

### Blood Samples

**Figure 1:**
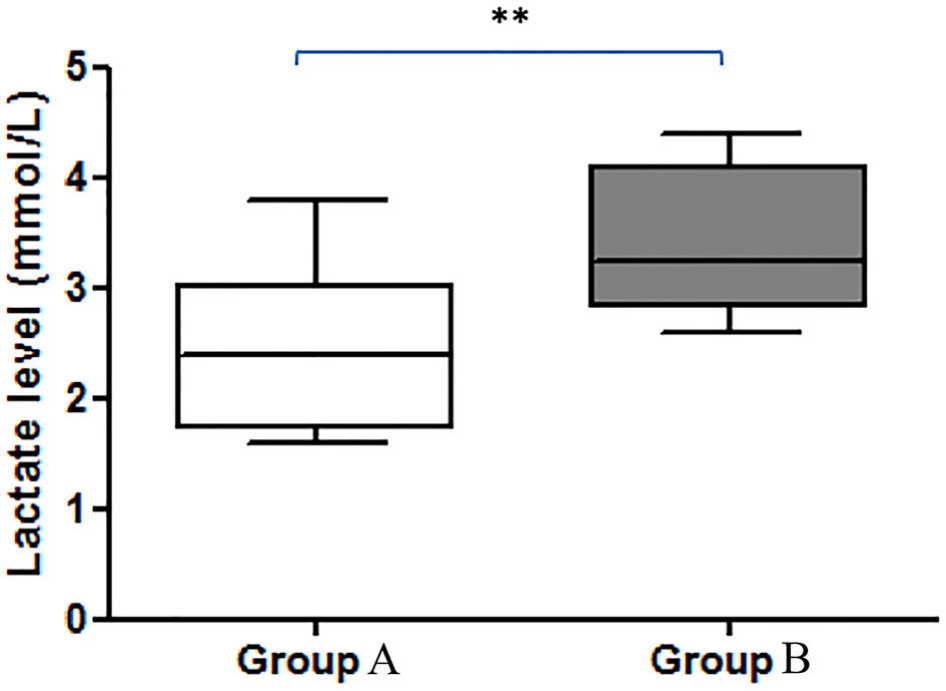
Comparison of the blood lactate levels (mmol/L) between groups. There was a significant difference between the increase in lactate in group A and group B (**p < 0.01). Group A included dogs with normal right ventricular function, and group B included dogs with impaired right ventricular function.

Blood samples were obtained from a cephalic vein, and the blood lactate concentration was measured with a blood lactate analyzer (blood lactate test meter; XPER Technology, Taiwan) before the echocardiography examination. Figure 1 shows the lactate values (mmol/L) of dogs in group 1 and group 2.

### Echocardiographic Examination

Dogs underwent transthoracic echocardiography [General Electric (GE) Medical System, vivid5s, Germany] to diagnose and observe disease progression. The measurement was performed when the dog was on lateral recumbence with no sedation. Echocardiographic images were captured and evaluated offline by only one sonographer. Left and right ventricular dimension and function were calculated from two-dimensional and M-mode images. The cardiac parameters were measured during diastole and systole, such as interventricular septum thickness during diastole (IVSd), interventricular septum thickness during systole (IVSs), left ventricular internal diameter during diastole (LVIDd), left ventricular internal diameter during systole (LVIDs), left ventricular proximal wall thickness during diastole (LVPWd), and left ventricular proximal wall thickness during systole (LVPWs). The diameter of the left atrium (LA) and aortic root (AO) ratio (LA/AO) were assessed at the level of the aorta when the aortic valves were closed during diastole.

**Figure 2:**
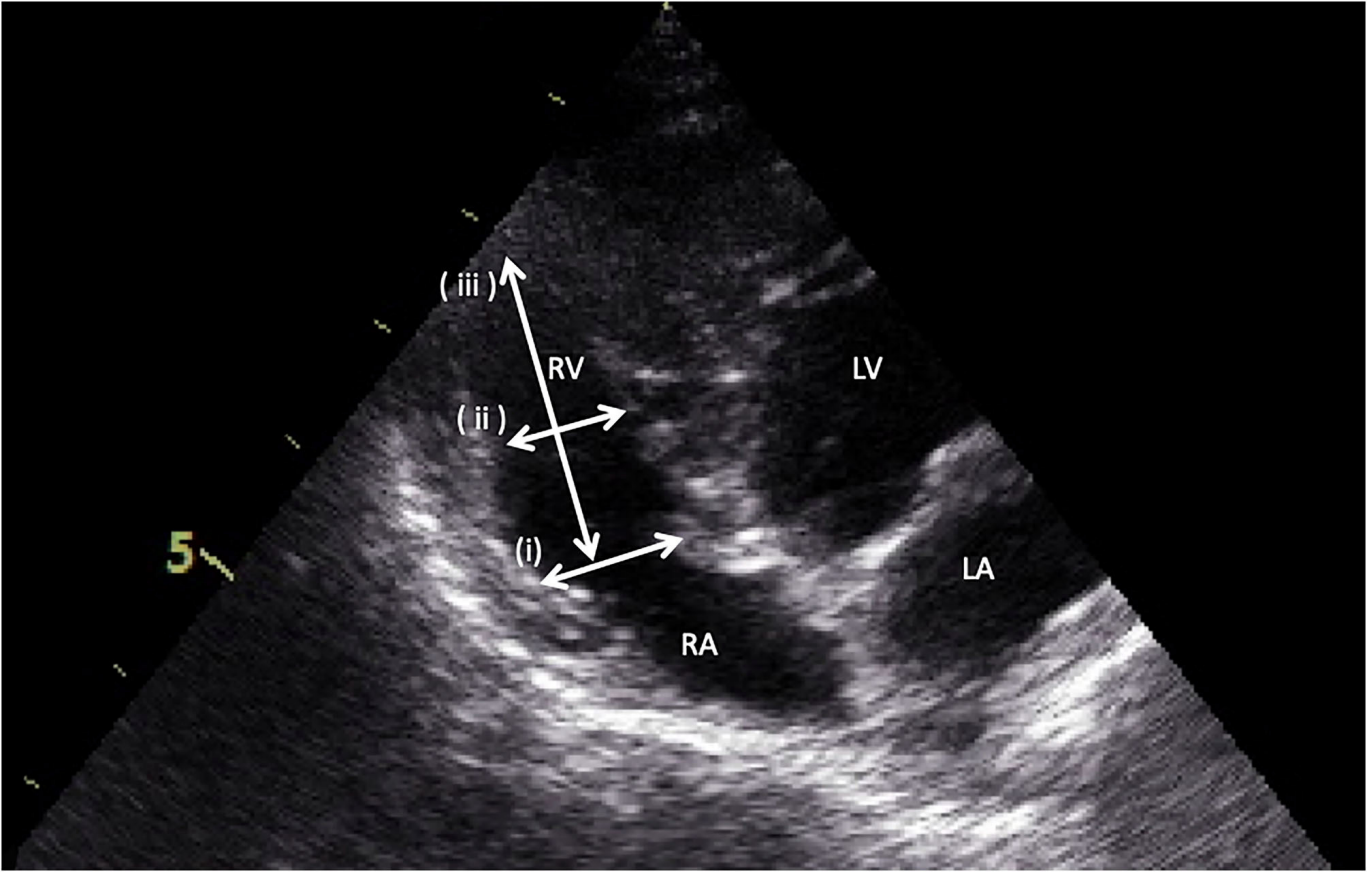
Measurement of right ventricular dimensions by the apical four-chamber view. (i) the basal distance measurement, (ii) the mid-right ventricular measurement, and (iii) the base to apex measurement.

**Figure 3:**
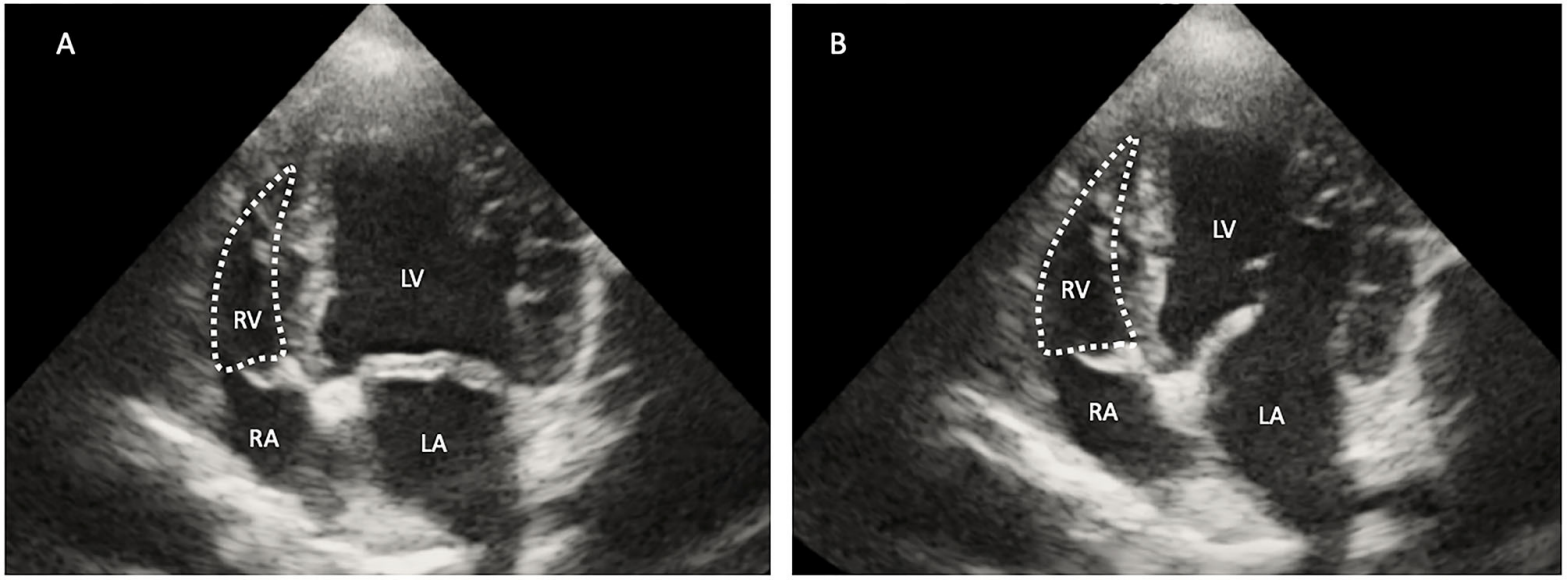
Two-dimensional echocardiographic image of the right ventricular area (RV) during systole **(A)** and diastole **(B)**. The right fractional area change (FAC) was calculated using the formula [(RV diastole – RV systole)/RV diastole] × 100. RA, right atrium; RV, right ventricle; LV, left ventricle; LA, left atrium.

Right ventricular dimensions were measured at end-diastole using the left apical 4-chamber view. Three sites are commonly used to determine the right ventricle size: (1) the basal distance measurement, (2) the mid-right ventricular measurement, and (3) the base to apex measurement, as shown in Figure 2. The right ventricular systolic function can be quantified using the fractional area change (FAC) of the right ventricle, which can be done on the apical 4-chamber view, as shown in Figure 3.

**Figure 4:**
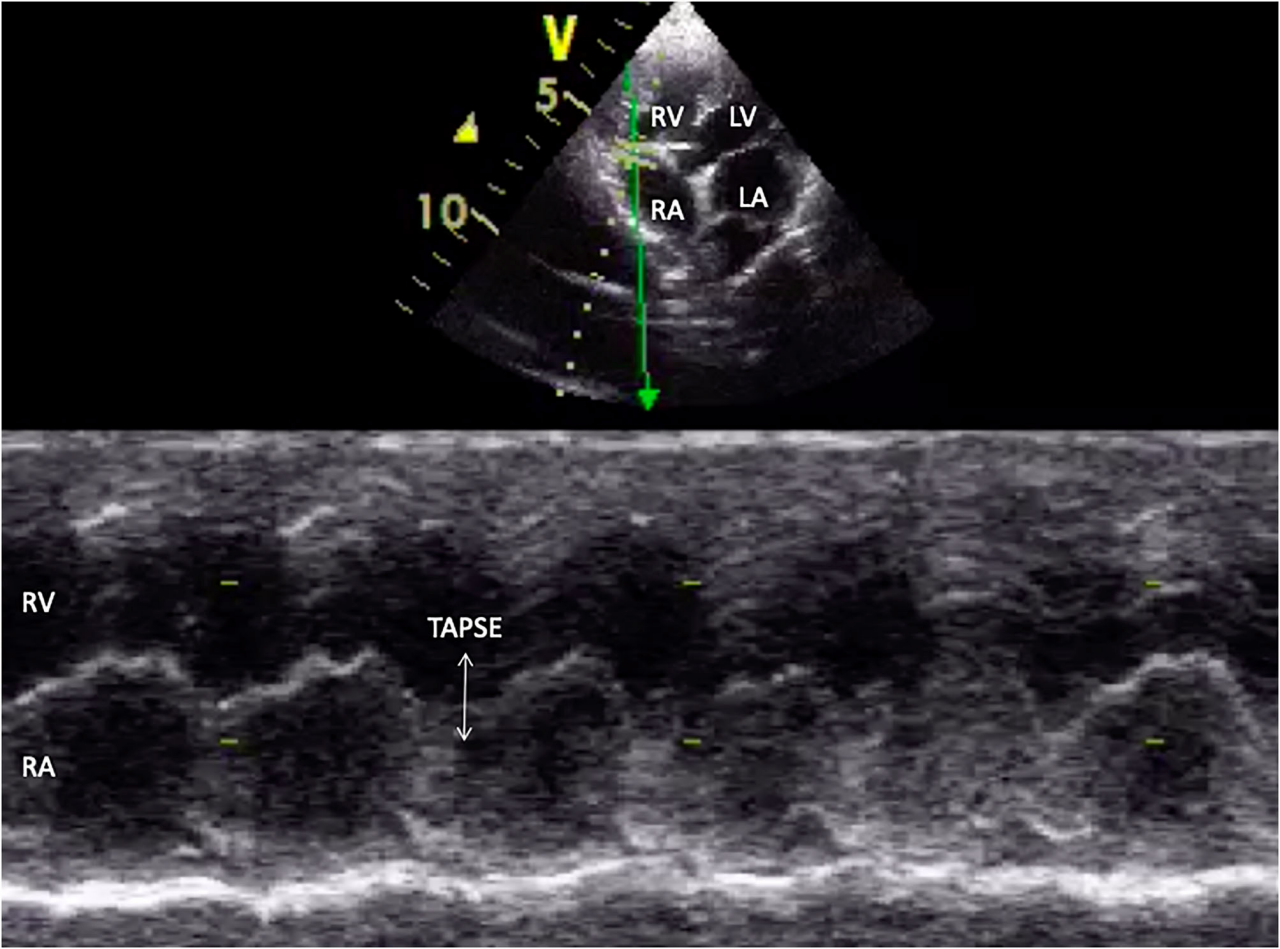
M-mode echocardiographic image of tricuspid annular plane systolic excursion (TAPSE) in appearance (white arrow). RA, right atrium; RV, right ventricle; LV, left ventricle; LA, left atrium.

Tricuspid annular plane systolic excursion (TAPSE) was evaluated by placing the M mode cursor through the tricuspid valve in the apical 4-chamber view, as shown in Figure 4.

Dogs were divided into two groups according to the presence of impaired right ventricular systolic function, which was defined as the presence of FAC <30%. Group A included dogs with normal right ventricular function, and group B included dogs with impaired right ventricular function.

### Analysis of Peptide Pattern by MALDI-TOF MS

The protein concentration in serum was determined by the Lowry method (23). The absorbance at 750 nm (OD750) was measured, and the protein concentration was calculated using the standard curve, plotted between OD750 on Y-axis and BSA concentration (g/ml) on X-axis. The peptides from serum were acidified with 0.1% trifluoroacetic acid to the final concentration of 0.1 mg/ml. The peptides were mixed with MALDI matrix solution (10 mg sinapinic acid in 1 ml of 50% acetonitrile containing 0.1% trifluoroacetic acid), directly spotted onto MALDI target (MTP 384 ground steel; Bruker Daltonik, GmbH), and dried at room temperature. Maldi-TOF MS spectra were collected using Ultraflex III TOF/TOF (Bruker Daltonik, GmbH) in a positive linear mode with a mass range of 2,000–15,000 Da. Five hundred shots were accumulated with a 50-Hz laser, and MS spectra were analyzed using flexAnalysis combination with ClinproTool software (Bruker Daltonik, GmbH), including fingerprint spectra, pseudo-gel view, and principal component analysis (PCA). The protein was calibrated using ACTH fragment 18–39 (human), Insulin oxidized B chain (bovine), Insulin (bovine), Cytochrome C (equine), and Apomyoglobin (equine). Data normalization and quantification of the changes in peptide abundance between the control and Maine Coon cats were performed and visualized using MultiExperiment Viewer (Mev) software version 4.6.1 (20). Briefly, peptide intensities from the LC-MS analyses were transformed and normalized using a mean central tendency procedure.

### Statistical Analysis

Paired *t*-test model was used to estimate and compare the parameters between two groups. Statistical analysis was performed using commercially available software (GraphPad Software Inc., USA). A value of *p* < 0.05 was considered statistically significant. The continuous variable showed a mean ± SEM. The survival analysis was analyzed using the Kaplan–Meier survival curve.

## Results

**Figure 5:**
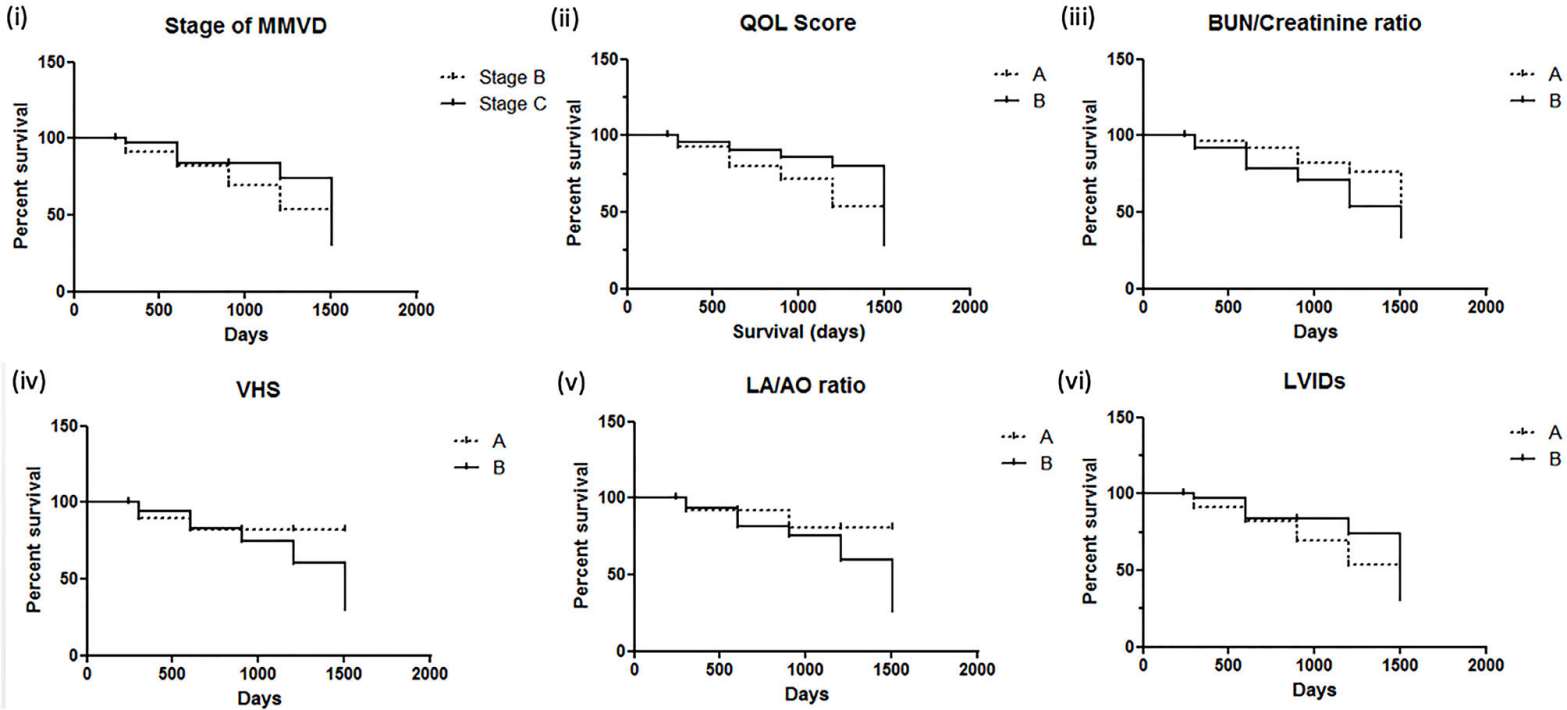
Kaplan–Meier survival curves for dogs with mitral valve degeneration. **(i)** Stage of MMVD (myxomatous mitral valve degeneration); **(ii)** QOL score, quality of life score (A = QOL score <3, B = QOL score >3); **(iii)** BUN/creatinine ratio (A = BUN/creatinine <20, B = BUN/creatinine >20); **(iv)** VHS, vertebral heart score (A = VHS <10.5, B = VHS >10.5); **(v)** LA/AO ratio, left atrium/aorta ratio (A = LA/AO <1.60, B = LA/AO >1.60); **(vi)** LVIDs, left ventricular internal diameter during systole (A = LVIDs <2.0, B = LVIDs>2.0). Group A included dogs with normal right ventricular function, and group B included dogs with impaired right ventricular function.

There were 93 dogs; at the end of the follow-up, 34 dogs had died due to their cardiac problems, and 59 dogs remained alive. The association between variable parameters and survival is shown in Figure 5. Kaplan–Meier curve shows the survival duration between group A and group B; Figure 5i displays the survival curve of MMVD patients with stage B and stage C. The cumulative probability of percent survival showed in MMVD patient stage B was 91.23, 82.54, 69.84, 53.40, and 37.38% for 1 to 5 years, respectively. In contrast, that of the patient in stage C was 96.87% and maintained at 83.51% for 2 and 3 years, and 74.23 and 30.13% for 4 and 5 years, respectively. The parameters such as vertebral heart score (VHS), left atrial and aorta diameter ratio (LA/AO), and quality of life score (QOL) had higher percent survival in group B than dogs in group A at 1 year after MMVD was diagnosed (Figures 5ii,iv,v) Hyperlactatemia (lactate concentration >2.5 mmol/L) was observed in 64% of dogs with mitral valve degeneration. Lactate concentrations were 2.90 ± 0.26 mmol/L in group A and 3.40 ± 0.23 mmol/L in group B dogs. There was a significant difference between the two groups. Dogs in group B had significantly higher ages (Table 2). However, no significant difference was found between the two groups regarding the left ventricular dimension and volume. The echocardiographic analysis revealed no statistically significant difference between the left ventricular end-diastolic and systolic volumes.

**Table 2:** Characteristics variables for two groups (*p < 0.05).

**Parameters**	**Group A**	**Group B**
Age in years	10.2 ± 0.82*	12.49 ± 0.63*
Male (%)	28	30

*Group A included dogs with normal right ventricular function, and group B included dogs with impaired right ventricular function*.

However, group B had a significantly higher incidence of left atrial and left ventricle dilation (Table 3). Group B showed significantly large right ventricular dimensions at the basal level and a worse degree of tricuspid regurgitation (TR) and lower fractional area change and TAPSE (Table 4). Right ventricular function in dogs with left-sided congestive heart failure is different between the stages of congestive heart failure. Dogs in ACVIM stage C showed decreased right ventricular systolic function as estimated by the decreased fractional area change and TAPSE relative to a previous study (12).

**Table 3:** Echocardiography evaluation for two groups regarding left ventricular echocardiographic findings.

**Parameters**	**Group A**	**Group B**	***P*-value**
Stage B (%)	40%	8%	–
Stage C (%)	18%	34%	–
LA/AO ratio	1.59 ± 0.05	1.95 ± 0.09	0.0007
LVIDd (cm)	2.72 ± 0.20	3.12 ± 0.14	NS
LVIDs (cm)	1.94 ± 0.18	2.31 ± 0.11	0.0826
EDV (ml)	33.96 ± 5.83	41.54 ± 4.34	NS
ESV (ml)	14.0 ± 3.70	20.41 ± 2.27	NS
E deceleration time (ms)	125.38 ± 2.61	90.08 ± 3.33	0.0001
FS (%)	32.0 ± 1.37	25.88 ± 0.75	0.0002

*Group A included dogs with normal right ventricular function, and group B included dogs with impaired right ventricular function*.

**Table 4:** Comparison between the two groups regarding right ventricular echocardiographic parameters.

**Parameters**	**Group A**	**Group B**	***P*-value**
RV basal diameter (cm)	1.10 ± 0.06	1.32 ± 0.08	0.0302
RV mid diameter (cm)	0.90 ± 0.05	0.95 ± 0.07	NS
RV long diameter (cm)	2.18 ± 0.10	2.35 ± 0.16	NS
FAC (%)	37.05 ± 1.56	22.38 ± 1.21	0.0001
TAPSE (cm)	1.31 ± 0.07	1.09 ± 0.07	0.0286
**TR [number (%)]**			
Mild	80	36	–
Moderate	20	64	–

*Group A included dogs with normal right ventricular function, and group B included dogs with impaired right ventricular function*.

**Figure 6:**
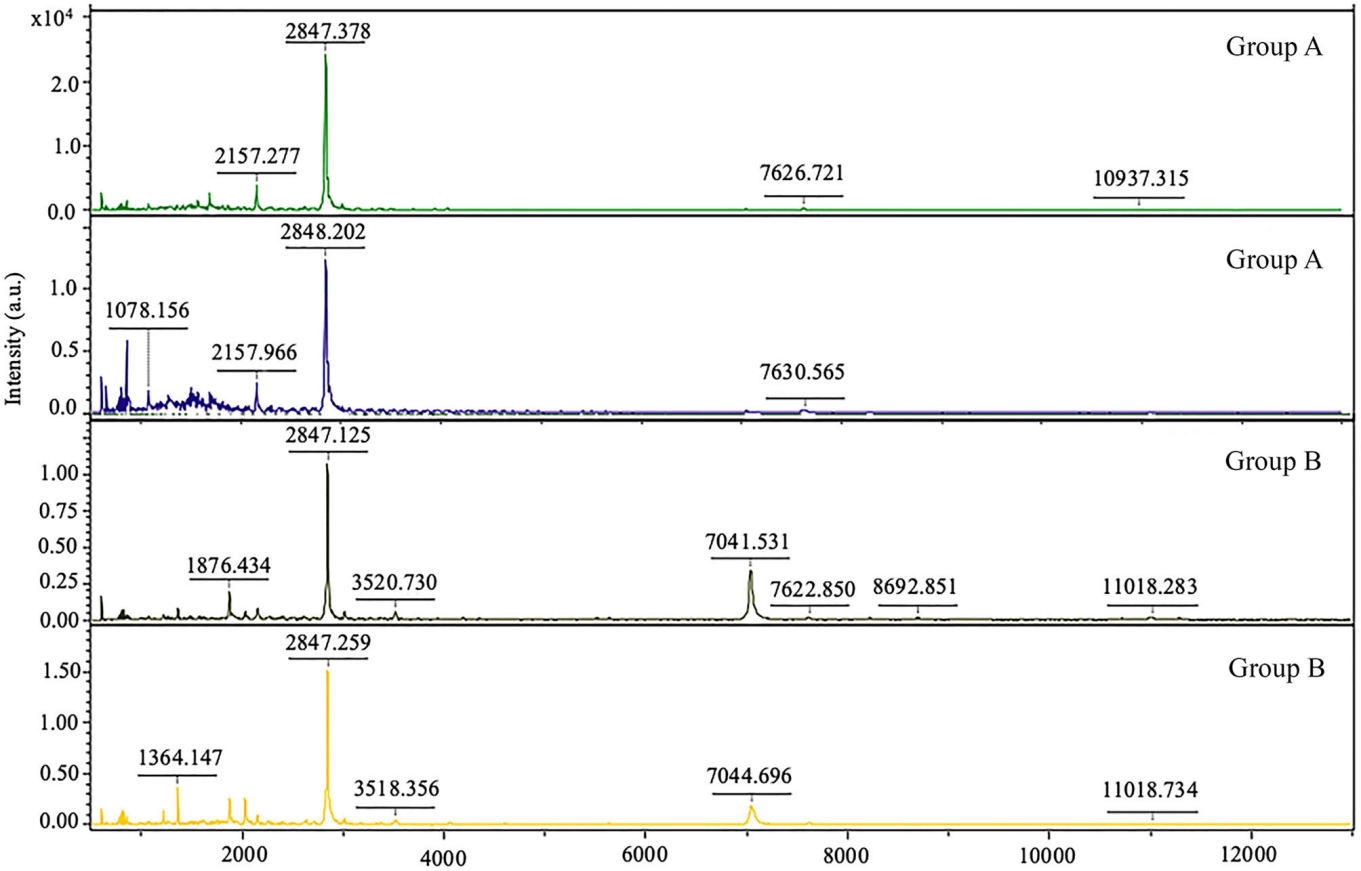
Peptide mass fingerprint of dogs with normal right ventricular function (group A) and dogs with impaired right ventricular function (group B).

In the present study, we aimed to examine the peptide complement of the serum isolated from control dogs (group A) and dogs with impaired right ventricular function (group B). For preliminary comparison of the expression levels of the peptide's profiles, peptide mass fingerprint (Figure 6) was used, and LC/MS analyzed the specific peptide sequences. The control groups (group A) and group B (dogs with impaired right ventricular function) were identified with a detection range of 1,000–20,000 Da. Figure 6 shows the identification of peptide mass and expected proteins. A mass spectral peak of peptide in our study showed that peptide fragments such as mitogen-activated protein kinase (MAPK), kallikrein related peptidase (KLK), and tenascin-C (TN-C) appeared in the heart disease progression group (group B).

## Discussion

This study's objective was to assess the prognostic information about valvular heart disease in dogs and investigate the right ventricular systolic function and blood lactate concentration. Dogs with clinical signs of congestive heart failure can be controlled, and they can live longer with better life quality by the appropriate medical treatment. Previous studies have reported the association with survival of echocardiographic variables such as left atrium and aorta ratio (LA/AO ratio), left ventricular internal diameter (LVID), and mitral E velocity. Our study also showed that heart size had an essential predictive value; left atrium dimension and vertebral heart score are associated with decreased survival in patients with degenerative mitral valve disease. However, the stage of mitral valve degeneration, systolic left ventricular internal diameter (LVIDs), and quality of life score were not indicators for survival predictors in our sample population. In addition, the percent survival in variables such as VHS, LA/AO ratio, and QOL was better in group B. Patients in group B contain more dogs in stage C, which had been treated with therapeutic drugs such as pimobendan, diuretic, and angiotensin-converting enzyme inhibitor. The results suggested that early treatment of MMVD patients in early stage B may improve the percent survival, which is similar to the ACVIM guideline (5).

The previous study reported the proteome expression in heart failure patients associated with the inflammation response and oxidative stress (19, 22), similar to our present study. The inflammation network response to mitral degeneration included the presentation of mitogen-activated protein 3 kinases (MAP3K), kallikrein related peptide (KLK), and tenascin C (TNC). Our data provided preliminary information on proteins in dogs with mitral valve degeneration. In addition, the peptidomics profiles may provide a potential independent marker for the detection of mitral valve disease progression. However, protein expression in our study was expressed not only on the heart but many pathological conditions, such as renal diseases, cancer, tissue injury, and inflammation. Thus, extending validation of the protein source may confirm the use of this protein for the prognostic and follow-up marker in the future.

Renal dysfunction can use as a neuro-humoral change in heart disease. Our study showed that the BUN/creatinine ratio impacts the predictive value of degenerative mitral valve disease in dogs. Dogs with mitral valve degeneration ACVIM stage C had higher blood lactate concentrations than dogs in stage B2. Although dogs in groups A and B were not found to have significant differences in the lactate values, the previous studies showed a significant difference between active vs. non-active subjects (24). Thus, it is essential to measure a dog's lactate values with similar physical activity before echocardiography.

This study suggested that the right and left ventricular functions are functionally related to each other. Right ventricular systolic dysfunction was relatively common in dogs with left-sided congestive heart failure, as 50% of the dogs in this study had right ventricular systolic dysfunction. Dogs with right ventricular systolic dysfunction had significant left atrium dilation. A worse ACVIM stage of congestive heart failure than dogs without right ventricular dysfunction was observed, as 34% of dogs in group B had ACVIM stage C, but 18% had it in group A. The current study demonstrated that less TAPSE was associated with left-sided systolic and diastolic dysfunction. The E deceleration time was significantly shorter in dogs with right ventricular dysfunction. An explanation for right heart dysfunction could be that the alterations in preload and afterload that occur secondarily to left-sided congestive heart failure may alter the right heart function described earlier (25, 26).

In discordance with the present study, dogs with right heart systolic dysfunction had no significant difference in end-systolic and end-diastolic dimensions or left ventricular internal diameter. Right ventricular imaging was considered a limitation of the present study. Right ventricular imaging in dogs with congestive heart failure is challenging due to the cardiac remodeling that could affect the echocardiographic measurement. Cardiovascular magnetic resonance imaging is recommended as the standard gold technique for the right-side assessment due to the complexity of the right-side heart anatomy.

## Conclusion

Dogs with mitral valve degeneration can have a mild increase in blood lactate values. In addition, dogs with right ventricular systolic dysfunction defined as FAC <30% and TAPSE <110 mm showed a significantly larger RV dimension at the basal level and a significantly worse degree of tricuspid regurgitation. Right ventricular dysfunction in degenerative mitral valve disease is common and is independent of left ventricular volume but is proportional to left contraction impairment. Peptidomics analysis results in this study suggested that these proteins may represent novel biomarkers of the disease progression in dogs with mitral valve degeneration. Our results in this present study will help in the detection and prognosis of mitral valve disease in dogs.

## Data Availability Statement

The datasets presented in this study can be found in online repositories. The names of the repository/repositories and accession number(s) can be found below: https://www.uniprot.org/.

## Ethics Statement

The study protocol was approved by the Ethics Committee, Kasetsart University (ACKU-62-VET-002). Written informed consent was obtained from the owners for the participation of their animals in this study.

## Author Contributions

SP wrote the original article, prepared the figures, and was a major contributor in writing the article. MY, MK, BI, and TP analyzed and interpreted the data. All authors contributed to the article and approved the submitted version.

## Conflict of Interest

The authors declare that the research was conducted in the absence of any commercial or financial relationships that could be construed as a potential conflict of interest.

## Publisher's Note

All claims expressed in this article are solely those of the authors and do not necessarily represent those of their affiliated organizations, or those of the publisher, the editors and the reviewers. Any product that may be evaluated in this article, or claim that may be made by its manufacturer, is not guaranteed or endorsed by the publisher.

## References

[1] Enriquez-Sarano M Akins CW Vahanian A. Mitral regurgitation. Lancet. (2009) 373:1382–94. 10.1016/S0140-6736(09)60692-9 19356795

[2] Lord P Hansson K Kvart C Häggström J. Rate of change of heart size before congestive heart failure in dogs with mitral regurgitation. J Small Anim Pract. (2010) 51:210–8. 10.1111/j.1748-5827.2010.00910.x 20406369

[3] Visser LC Scansen BA Schober KE Bonagura JD. Echocardiographic assessment of right ventricular systolic function in conscious healthy dogs: repeatability and reference intervals. J Vet Cardiol. (2015) 17:83–96. 10.1016/j.jvc.2014.10.003 25547662

[4] Atkins C Bonagura J Ettinger S Fox P Gordon S Haggstrom J. Guidelines for the diagnosis and treatment of canine chronic valvular heart disease. J Vet Intern Med. (2009) 23:1142–50. 10.1111/j.1939-1676.2009.0392.x 19780929

[5] Keene BW Atkins CE Bonagura JD Fox PR Haggstrom J Fuentes VL. ACVIM consensus guidelines for the diagnosis and treatment of myxomatous mitral valve disease in dogs. J Vet Intern Med. (2019) 33:1127–40. 10.1111/jvim.15488 30974015 PMC6524084

[6] Iwanuk N Wall L Nolte I Raue J Rumstedt K Pilgram A. Effect of pimobendan on physical fitness, lactate and echocardiographic parameters in dogs with preclinical mitral valve disease without cardiomegaly. PLoS ONE. (2019) 14:e0223164. 10.1371/journal.pone.0223164 31581204 PMC6776412

[7] Fahey R Rozanski E Paul A Rush JE. Prevalence of vomiting in dogs with pericardial effusion. J Vet Emerg Crit Care. (2017) 27:250–2. 10.1111/vec.12570 28079972

[8] Le Tourneau T Deswarte G Lamblin N. Right ventricular systolic function in organic mitral regurgitation: impact of biventricular impairment. Circulation. (2013) 127:1597–608. 10.1161/CIRCULATIONAHA.112.000999 23487435

[9] Kjaergaard J Iversen KK Akkan D. Predictors of right ventricular function as measured by tricuspid annular plane systolic excursion in heart failure. Cardiac Ultrasound. (2009) 7:51. 10.1186/1476-7120-7-51 19889228 PMC2776003

[10] Chapel EH Scansen BA Schober KE Bonagura JD. Echocardiographic estimates of right ventricular systolic function in dogs with myxomatous mitral valve disease. J Vet Intern Med. (2018) 32:64–71. 10.1111/jvim.14884 29224256 PMC5787149

[11] Haddad F Hunt SA Rosenthal DN Murphy DJ. Right ventricular function in cardiovascular disease, part I: anatomy, physiology, aging, and functional assessment of the right ventricle. Circulation. (2008) 117:1436–48. 10.1161/CIRCULATIONAHA.107.653576 18347220

[12] Desai RV Meyer P Ahmed MI. Relationship between left and right ventricular ejection fractions in chronic advanced systolic heart failure: insights from the BEST trial. Eur J Heart Fail. (2011) 13:392–7. 10.1093/eurjhf/hfq206 21097899 PMC3063564

[13] Ueda Y Gunther-Harrington CT Cruzen CL Roberts JA Stern JA. Echocardiographic parameters of clinically normal geriatric rhesus macaques (Macaca mulatta). J Am Assoc Lab Anim Sci. (2017) 56:361–8. PMC5517324 28724484

[14] Lang RM Badano LP Mor-Avi V Afilalo J Armstrong A Ernande L. Recommendations for cardiac chamber quantification by echocardiography in adults: an update from the American society of echocardiography and the European association of cardiovascular imaging. J Am Soc Echocardiogr. (2015) 28:1–39. 10.1016/j.echo.2014.10.003 25559473

[15] Bleeker GB Steendijk P Holman ER. Assessing right ventricular function: the role of echocardiography and complementary technologies. Heart. (2006) 92:i19–26. 10.1136/hrt.2005.082503 16543597 PMC1860734

[16] Rudski LG Lai WW Afilalo J Hua L Handschumacher MD Chandrasekaran K. Guidelines for the echocardiographic assessment of the right heart in adults: a report from the American society of echocardiography. J Am Soc Echocardiogr. (2010) 23:685–713. 10.1016/j.echo.2010.05.010 20620859

[17] Geyer PE Kulak NA Pichler G Holdt LM Teupser D Mann M. Plasma proteome profiling to assess human health and disease. Cell Syst. (2016) 2:185–95. 10.1016/j.cels.2016.02.015 27135364

[18] Egerstedt A Berntsson J Smith ML. Profiling of the plasma proteome across different stages of human heart failure. Nat Commun. (2019) 10:5830. 10.1038/s41467-019-13306-y 31862877 PMC6925199

[19] Zarubin T Han J. Activation and signaling of the p38 MAP kinase pathway. Cell Res. (2005) 15:11–18. 10.1038/sj.cr.7290257 15686620

[20] Yin H Chao L Chao J. Kallikrein/kinin protects against myocardial apoptosis after ischemia/reperfusion via Akt-GSK-3 and Akt-Bad-14-3-3 signaling pathways. J Biol Chem. (2005) 280:8022–30. 10.1074/jbc.M407179200 15611141

[21] Yao YY Yin H Shen B Chao L Chao J. Tissue kallikrein infusion prevents cardiomyocyte apoptosis, inflammation and ventricular remodeling after myocardial infarction. Regul Pept. (2007) 140:12–20. 10.1016/j.regpep.2006.11.020 17196272 PMC1876786

[22] Imanaka-Yoshida K. Tenascin-C in heart diseases: the role of inflammation. Int J Mol Sci. (2021) 22:5828. 10.3390/ijms22115828 34072423 PMC8198581

[23] Lowry OH Rosebrough NJ Farr AL Randall RJ. Protein measurement with the folin phenol reagent. J Biol Chem. (1951) 193:265–75. 10.1016/S0021-9258(19)52451-6 14907713

[24] Restan AZ Camacho AA Cerqueira JA Zacche E Kirnew MD Loureiro BA. Effect of a lactate-guided conditioning program on heart rate variability obtained using 24-Holter electrocardiography in Beagle dogs. PLoS ONE. (2020) 15:e0233264. 10.1371/journal.pone.0233264 32479554 PMC7263627

[25] Yuchi Y Suzuki R Kanno H Teshima T Matsumoto H Koyama H. Right ventricular myocardial adaptation assessed by two-dimensional speckle tracking echocardiography in canine models of chronic pulmonary hypertension. Front Vet Sci. (2021) 8:727155. 10.3389/fvets.2021.727155 34485446 PMC8415444

[26] Emilsson K. Right ventricular long-axis function in relation to left ventricular systolic function. Clin Physiol Funct Imaging. (2004) 24:212–5. 10.1111/j.1475-097X.2004.00550.x 15233835

